# SIAL: A simple image analysis library for wet-lab scientists

**DOI:** 10.21105/joss.02689

**Published:** 2020-12-14

**Authors:** David R. Tyrpak, Yaocun Li, Siqi Lei, J. Andrew MacKay

**Affiliations:** 1 Department of Pharmacology and Pharmaceutical Sciences, School of Pharmacy of the University of Southern California; 2 Keck School of Medicine of the University of Southern California, Department of Ophthalmology, Roski Eye Institute; 3 University of Southern California Viterbi School of Engineering, Biomedical Engineering

## Summary

In many biomedical research labs, image analysis tasks are relatively simple but labor intensive. For example, a typical workflow may require human intervention to outline regions of interest or score phenotypes from a collection of images. Such tasks could potentially be partially automated with a script or machine learning, but in our experience, many biomedical researchers do not obtain programming skills. Furthermore, image analysis is typically just one of the many experiments that a busy researcher will employ during the course of a project, and so devoting time to learning programming or bespoke image analysis is not prioritized. These factors create an unfortunate situation where, in addition to being labor intensive, image analysis workflows may become biased and exhibit limited reproducibility.

As primarily “wet-lab” scientists, we wanted to develop user friendly programs focused on some of the most common tasks that fellow wet-lab researchers encounter during image analysis. Because FIJI/ImageJ (Schindelin et al., 2012) is routinely used by biomedical researchers the world over, we developed our programs in Java and packaged them together as a FIJI plugin. We name this plugin SIAL: A simple image analysis library. SIAL aids users in human-assisted image analysis by providing programs for image pseudonymization, phenotype scoring, and ROI harvesting. Each of the three programs inside SIAL is briefly summarized below:

### File Randomizer:

This program provides a convenient way for researchers to pseudonymize their imaging data, which is a critical step in any analysis involving phenotype scoring or ROI selection. The program copies images in a specified input directory to a specified output directory, and then randomly renames the copied images with a three-digit number. It also outputs a key matching the original filenames to the renamed images and a details file which records time of analysis, as well as the specified input and output directory.

### PhenoScoreKeeper:

This program helps speed up manual phenotype scoring of images by partially automating score collection. Users specify an input directory and output directory, and the program automatically opens up each image in the input directory and prompts the user to enter an integer score for the image. It saves the scores in a csv file located in the specified output directory.

### ROI Recorder:

This program uses ImageJ’s built-in ROI manager to quickly harvest ROIsets and measurements from images in a specified input directory. The program opens up the input images one at a time and prompts the user to harvest ROIs. It then automatically saves the ROIsets and ROI measurements for each image to the specified output directory.

Both the PhenoScoreeKeeper and ROI Recorder programs track which images have already been analyzed so that users can stop and start their analyses without losing progress. Links to videos detailing the use of these programs are provided in the Installation and Tutorials section.

## Statement of need

Many biomedical researchers perform relatively simple image analysis tasks that often require human intervention. Notable examples include scoring phenotypes or harvesting ROIs from a directory of images. FIJI already provides many of the tools to carry out these analyses, but workflows are often compromised by user bias and limited reproducibility, as users may analyze data without proper blinding or neglect to save useful metadata. In addition, even these simple analyses can become labor intensive in the absence of a script which automates repetitive tasks while saving relevant output.

SIAL’s goal is to provide simple GUI programs to help wet-lab researchers blind and pseudonymize their data, score phenotypes, and harvest ROIs. In addition, because wet-lab scientists are typically interrupted by multiple experiments, the PhenoScoreKeeper and ROI Recorder programs keep track of which images in a directory have already been analyzed so that researchers can easily start and stop their workflows without hassle.

SIAL is easily installed via the ImageJ update website service, utilizes simple user interfaces, requires no programming experience, and requires no dependencies except FIJI. The individual programs inside SIAL can be easily integrated with workflows involving other FIJI plugins or with workflows employing another open source software, like Cell Profiler (McQuin et al., 2018) or QuPath (Bankhead et al., 2017). For example, users may employ the File Randomizer to blind their data before downstream analyses with these programs. Alternatively, within FIJI users may harvest ROIs using the ROI Recorder program and then analyze the ROIs using another plugin or import the ROIsets into QuPath for complementary analysis.

Because of its ease of use and focus on some the most common wet-lab image analysis tasks, SIAL is routinely used by our group and our close collaborators to improve the efficiency and reproducibility of our image analysis workflows.

## Supplementary Material

Zip file of software

## Footnotes

Installation and Tutorials First ensure you have FIJI installed on your computer: https://fiji.sc To download SIAL, open FIJI, go to “Help > Update…” and then update FIJI. After FIJI is finished downloading all updates, a window named “ImageJ Updater” will open. Select “Manage Update Sites > Add update site” and add this url: https://sites.imagej.net/D-tear/ Be sure to check the box next to this update site to ensure the FIJI adds SIAL to your FIJI Plugins folder. Select “Close > Apply changes.” FIJI will download SIAL.jar and associated dependencies. After successfully updating, FIJI will then ask to be closed and restarted. After doing this, SIAL can be accessed via the Plugins tab in FIJI. Note that SIAL will usually be installed towards the bottom of the available FIJI plugins. Links to YouTube tutorials covering the installation and use of SIAL: Installing SIAL: https://youtu.be/RU4B3GhVwaM How to use the File Randomizer program inside of SIAL: https://youtu.be/_oQLJ5gC7ls How to use the PhenoScoreKeeper inside of SIAL: https://youtu.be/FiaNubIDvSw How to use the ROI Recorder program inside of SIAL: https://youtu.be/9mTHGVeWJA0 How to import FIJI ROIsets into QuPath: https://youtu.be/hCFrk9NJ4gk
